# Out-of-hours GPs and palliative care-a qualitative study exploring information exchange and communication issues

**DOI:** 10.1186/1472-684X-9-18

**Published:** 2010-08-12

**Authors:** Mark Taubert, Annmarie Nelson

**Affiliations:** 1 Palliative Care Department, Cardiff University, Velindre Hospital, CF14 2TL, Cardiff, UK; 2 Marie Curie Senior Research Fellow, Wales Cancer Trials Unit, Neuadd Meirionnydd, University Hospital of Wales, Heath Park, Cardiff, CF14 4YS, UK

## Abstract

**Background:**

Out-of-hours general practitioners (GPs) cover the community over a significant proportion of a given week, and palliative care patients are seen as a priority. Little is known about how well these GPs feel supported in their line of work and whether communication exchanges work well for the proportion of their patients who have palliative care needs. For this study, GPs who provide out-of-hours care were interviewed in order to explore factors that they identified as detrimental or beneficial for good communication between themselves, patients, relatives and other professionals, specifically to palliative care encounters.

**Methods:**

Nine GPs were interviewed using face-to-face semi-structured interviews. All nine GPs worked regular out-of-hours sessions. Data from transcripts was analysed using Interpretative Phenomenological Analysis.

**Results:**

A predominant theme expressed by GPs related to constraints within the system provided by the local private company owned out-of-hours provider. A strong feeling of 'being alone out there' emerged, with some GPs more willing to call for help than others, and others expressing their concern at access to pharmacies and medication being very inconsistent.

Out-of-hours GPs felt left alone on occasion, unable to access daytime services and not knowing who to call for advice. Information hand-over systems from in-hours to out-of-hours with regard to palliative care were felt to be inadequate. Out-of-hours doctors interviewed felt left out of the care loop; handover sheets from specialist palliative care providers were a rarity.

**Conclusions:**

Out-of-hours services need to be mindful of the needs of the GPs they employ, in particular relating to the palliative care they provide in this setting. Other healthcare professionals should aim to keep their local out-of-hours service informed about palliative care patients they may be called to see.

## Background

Out-of-hours service provision in the United Kingdom is defined as evenings, weekends from Friday evening to Monday morning, as well as public holidays [1]. These services are provided by General Practitioners, who at the time this study was undertaken had to, as a requirement to work in the out-of-hours setting, be registered on a local primary care trust (PCT) or local health board (LHB) performers list. The performers list system gives trusts and health boards across the United Kingdom responsibility for monitoring performance and conduct of general practitioners, with admission, suspension and removal procedures intended to parallel those for doctors in secondary care [2]. GPs wishing to work for the service are employed on a salaried basis and work in the setting either full-time or part-time [3].

Estimates suggest that palliative care patients with cancer spend 90% of their last year of life in their home [4], at least two-thirds of which will fall within out-of-hours-time [5]. The National Institute for Health Research highlighted the need to identify factors that improve and hinder the delivery of optimum palliative out-of-hours care in 2007 [6]. Programs underpinning end of life care policy include the End of Life Care Strategy, which is supporting the wider uptake of three initiatives: the Gold Standards Framework, the Liverpool Care Pathway, and Preferred Place of Care plans to enhance the choice of dying at home if patients wish [7-13]. The Scottish Partnership for Palliative Care has also made recommendations for general palliative care services and care of the dying [14] and the Northern Ireland Palliative Care Strategy Document similarly focuses on the need for wider roll-out of the existing three initiatives listed above [15].

Since the introduction of the new out-of-hours (OOH) provisions under the General Practitioner Contract in October 2004 [16,17] this area of care has changed drastically and a large percentage of the overall general practitioner workforce no longer works in this specific setting [18]. Many daytime family doctor practices handed over responsibility for providing out-of-hours to their local primary care organisation, in return for giving up an average of £6000 per year [19]. Local primary care organizations were then free to put the service out to tender and private companies took over out-of-hours provision in parts of the United Kingdom. There has been a shift from out-of-hours care provided by local general practices and their co-operatives to more distant emergency clinics or telephone advice [20]. The introduction of the new contract and the reconfiguration of out-of-hours services have altered the activities of GPs, and may affect their role in the provision of palliative care [21,22].

For specialist palliative care in Wales, a 24 hour/7 day (24/7) working pattern was part of the recommendations of the 'Sugar Report' in 2008 [23]. The report's aim was 'to ensure fairness of access to appropriate consultant-led specialist palliative care team support throughout Wales, in order to meet the needs of patients, their care-givers and professional colleagues.' In addition the National Cancer Standards (2005) require that palliative care needs should be rapidly addressed, and specialist palliative care advice be available, in all settings, twenty-four hours a day and this is set out in a Welsh Assembly document [24]. The Sugar report stated that 'The current way of working means much of the out-of-hours services are delivered by staff who lack the relevant skills. Out-of-hours support should be delivered by General Practitioner or District Nursing staff who have been sufficiently trained in palliative care and have access to 24/7 specialist support and advice. This must also include access to medication at all times.'[23].

**Figure 1:**
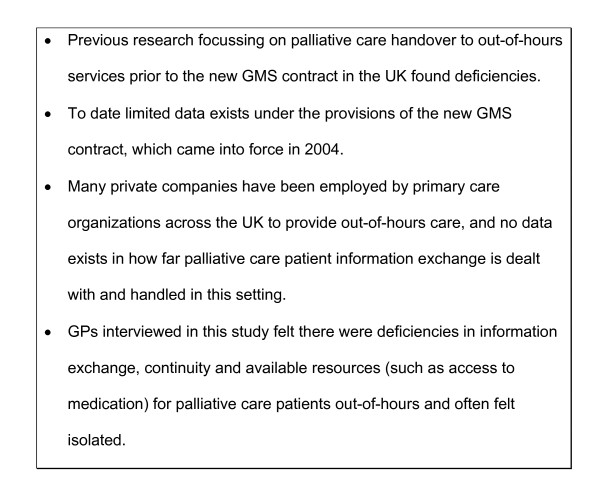
Summary

Furthermore the Welsh 'Implementation of Palliative Care Report Palliative Care Services funding 2008 to 2009' stated that 'It is a responsibility of specialist palliative care services to drive up standards of end-of-life care through education, both formal and informal, of other services and through support to other clinicians that is focused around meeting patient need.' [25]. This research project was set up to establish what GPs who work in the out-of-hours service feel about the organizational and communicational aspects of palliative care provision within the setting (Figure 1).

## Methods

### Local context

There is an allocated out-of-hours care provider for every primary care organization in the United Kingdom. In Cardiff, until October 2008 this was covered by a private company called Serco [26]. Serco is a FTSE 100 listed company and has experience in organizing out-of-hours services within other health boards across the United Kingdom, aiming to work alongside the NHS to improve community-based health services. At the time of the interviews in mid 2008, they provided out-of-hours GP services to more than one million people in Cardiff, Oxford and Cornwall handling calls from the public, running clinics and providing home visits to urgent cases. In 2010, Serco employed over three hundred doctors and nurses providing clinical services to over two million people in a range of primary and community settings. Apart from out-of-hours care provision, Serco delivers healthcare in prisons, nursing support as well as many major new health initiatives.

### Ethics approval

This study received ethics approval from South East Wales Research Ethics Committee

### Participant selection

A total of sixty doctors employed by Serco's out-of-hours service in Cardiff were contacted. To ensure sufficient out-of-hours exposure, General Practitioners who had worked in the out-of-hours setting for at least one year were chosen. Nine GPs came forward. No further recruitment drive was undertaken following the interviews and this is explained in the discussion section of this article.

### Participants

Five female and four male doctors agreed to be interviewed. Ages ranged from 28 to 58 years of age. All GPs worked in-hours shifts as well, either as a GP partner (n = 3), salaried GP (n = 3) or locum GP (n = 3). One GP had done the diploma in palliative medicine at Cardiff University and done a few sessions in the local hospice, but none of the other GPs had worked in specialist palliative care. GPs varied in how often they worked out-of-hours shifts between 2 and 12 sessions per month.

### Data collection

**Figure 2:**
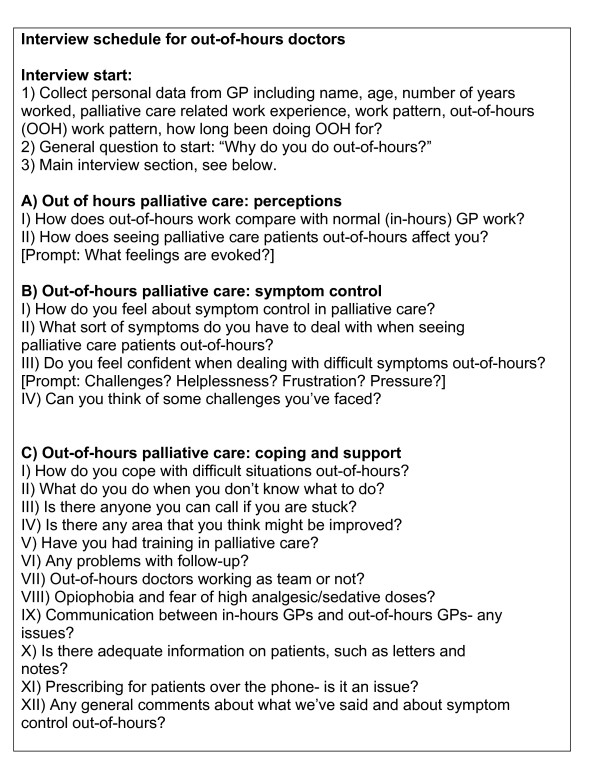
Interview Schedule

Semi-structured interviews were undertaken and audio-taped and field-notes were taken. The final interview schedule is given in figure 2. Interview length ranged from 39 minutes to 1 hour 21 minutes and the average duration was 58 minutes. The interviews were conducted face-to-face in the interviewees place of choice, usually place of work (office) or the home setting. MT was the interviewer for all nine participants. Interviews were subsequently transcribed verbatim.

## Data analysis

**Figure 3:**
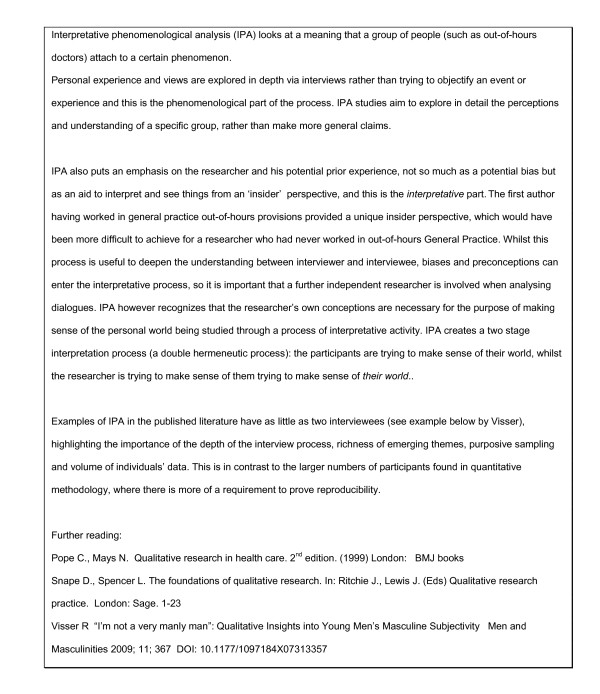
Choice of research method (Interpretative Phenomenological Analysis)

Two investigators analysed transcripts using Interpretative Phenomenological Analysis (IPA) (Figure 3). IPA research seeks to "make sense of the participant making sense of their own world". At the same time, it allows the researchers to interpret the participant's perspectives [27,28].

Transcripts were read and re-read by both investigators to increase reliability and during this process, important comments and reflections were highlighted if they were deemed to be relevant to the overall theme. Comments were categorized into themes by one of the researchers (MT) and given codes. A second researcher (AN) subsequently re-read transcripts and either added or grouped topics utilizing the same coding system. During the process, researchers paid attention to the relationship between language use and content of speech. This included choice of vocabulary participants used and the use of metaphors. Both researchers then agreed upon master themes and sub-categorized individual themes accordingly. Cross-over of themes between interviews was also checked for.

## Results

**Figure 4:**
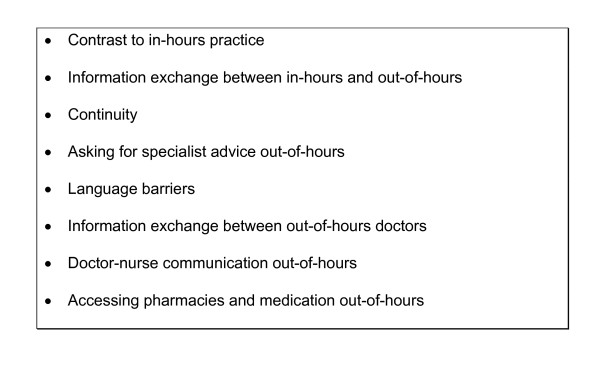
Subthemes found during analysis

Interviews were thematically rich in comments about how information gets exchanged within out-of-hours, how this affected continuity and what resources were or were not available (Figure 4). The results presented here pertain specifically to what GPs said about information exchange, communication and services. A further study, based on these nine interviews, has been published and focuses on how out-of-hours doctors perceive themselves within the domain of palliative care [29].

### Contrast to in-hours practice

All GPs interviewed for this project also worked as in-hours GPs. They commented about the contrasts to what happens in in-hours practice, where patients are followed up. GPs commented on the singularity of out-of-hours encounters, and one likened it to a 'snapshot', expressing the brevity of the encounters and how they lacked the prior knowledge that an in-hours GP might have of a patient.

GP 1 ...if you meet them in out-of-hours it's a snapshot of a particular day...

Unpredictability and perceived lack of control were areas highlighted by GP7.

GP7 Quite unstructured, quite disorganised, are perhaps the adequate phrases to describe, can't plan for it just have to deal with the things and hope it is ok.

### Information exchange between in-hours and out-of-hours

When it came to information exchange between the in-hours service and the out-of-hours service, there was a perceived problem by all GPs interviewed. GP6 and GP7 had ideas on how things should work, thinking it should be made compulsory for in-hours GPs to pass on their palliative patients to out-of-hours and keep good notes. Both used very definitive words in this context, *compulsory *and *obliged*, indicating that an authoritative intervention in their views was necessary to get to this (improved) level of functioning.

GP6 ...and that if someone is palliative that at least out-of-hours is informed about it (...) if someone is put on the palliative care pathway, it should be compulsory to pass that on to out-of-hours.

GP7 The only thing, you know it would be good if you could communicate with your professional colleagues, you know with a care plan, what the GPs, where the GPs in-hours are obliged to write notes, the Macmillan nurse also are obliged to write notes, and then leave them at patient's house. You have to have the notes. And to be obliged to do that.

Lack of planning from the in-hours GP was also felt to increase the workload in out-of-hours.

GP4 It would be great, errm. It doesn't happen that often. It really doesn't. And if the in-hours realised how much work and confusion could be saved by just sending a one-page summary or something like a letter.

GP8 felt strongly that out-of-hours workload in palliative care could be improved by a specific plan from in-hours services.

GP8 ...I think palliative patients as much as possible should have a, plan for sort of the worst case scenario out-of-hours so that everyone knows what to do. Because usually what happens is that the worst case scenario happens and there's no planning in place and you're the one who has to sort it all out somehow.

Similarly GP6 felt strongly that on several occasions he had been left in the lurch by the in-hours GP.

GP6 Then they phone up, the relatives on a Saturday and they've run out of medication. That's happened to me before, and you're there and what do we do?

Asked how he feels about this he responds strongly and aggressively.

GP6 Pissed off! (silence 1 second) Yeah, because, you think, this guy hasn't done his job properly, and, and, and, now, now, now I'm stuck with this patient. Bloody great!

### Continuity

More than half of the interviewed GPs, in particular GP2, commented on difficulties with continuity due to the particular work system in out-of-hours. This was, they felt, the result of short shifts within the rota system and this meant it was tempting to pass the patient on to the next shift or the in-hours GP. But he stated that he felt able to move on and then forget about the situation later. Given how well he remembered the lady in the above encounter, one could question whether he *does *forget that easily.

GP2 ..then you move on and forget. It's just a night done.

Other GPs made similar comments about forwarding on care to another GP, and being able to move on quickly.

GP3 If you're not comfortable with what's going on, you can forward it on to their own GP on the Monday (laughs).

GP9 ...to say to them 'call back later if you're still having problems'. By which point you're off the shift for another week and you won't ever see them again.

This had not always been the case in out-of-hours care, as recounted by GP5, who reminisced about being able to follow-up as an out-of-hours doctor.

GP5 Yeah, follow-up, you can't do that, when I've worked in the past Saturday and Sunday mornings out-of-hours, so I could easily say 'look I'm going to be back on tomorrow and I can do it' but I think we're losing an awful lot of that now, it's not like it used to be.

On the subject of continuity and on a similar vein as the one above (short shift patterns), a major recurring theme within interviews was lack of ownership, which most saw as a disadvantage.

GP4 ...you've got a much higher level of, errm, ownership..(in-hours GP compared to out-of-hours GP)

GP4 ...there isn't the facility to chase it up with a phone-call...you lose interest.

Nearly all GPs felt strongly that there should be some written information at patients' homes, in the form of letters or notes.

GP6 ...patient details should be more easily available. Ideally the in-hours GP notes or a print-out summary at the patient's home, or some letters from the oncologists or whoever.

Two GPs in particular expressed concerns about the out-of-hours record-keeping system.

GP6 A bit crap. But I don't know how you could change that. Maybe it would need a modern, a better record-keeping system, or a system where the out-of-hours docs have access to all in-hours GP-note systems, but that is quite utopian (laughs).

GP7 But currently facilities for recording out-of-hours consultations is very haphazard.

### Asking for specialist palliative care advice out-of-hours

When it came to asking for help with palliative care patients, there were differences in opinion. Some felt confident to ask for specialist palliative care advice and had had good experiences, even comparing with other specialties that were less helpful.

GP4 And generally I find that Oncology and Palliative Care docs do seem to be quite sympathetic to GPs and to SHOs [junior hospital doctors] as well, who ring them up for advice, I think they realise that we sometimes probably struggle with some of these things, errm, and they're not usually snappy like surgeons can be.

GP2 initially 'thinks aloud' about calling for specialist palliative care advice.

GP2 You know what to do, but you still kind of felt you might have to ring someone about it, I suppose wanting to know are you doing the right thing here?

But from his next sentence one can deduce that the he is not at all confident about calling specialist palliative care and this may be due to lack of confidence and fear of an unknown entity (in this case the on-call specialist) and even potential confrontation.

GP2 ...and I suppose as doctors we're also human beings, we like to know that the situation's a familiar one and not one of a long pause on the other end of the line so it's 'I really don't know who this is and what you're talking about in this case'. And that is reflected in, you know, the tone and you know, voice of whoever you might be speaking to. I think these are all important parts of the phone conversation.

It also became clear during this interview that the GP did not actually know the palliative care on-call structure, i.e. had not ever used the service, and this was the case for some of the other GPs interviewed also. Did GP2 not use the facility of palliative care specialist advice because he feared, as he had expressed 'a long pause on the other end of the line' and did this mean he generally didn't call on specialist telephone advice in general?

GP2 I haven't actually felt pushed to contacting somebody out-of-hours, I'm not aware they might be, although even if there was an opportunity where I would have to, I'd maybe go through the main local hospitals...

MT: What lowers that confidence in the out-of-hours situation?

GP2: The fact that, it is highly likely that I may not be able to speak to someone on a palliative team.

The above statement shows a lack of knowledge of on-call structures, as there is locally in Cardiff a middle grade doctor and a consultant on-call at all times including weekends and this is via the local hospice rather than local hospital switchboards.

The language he uses in his account is full of a sense of limits of his own agency, full of uncertainty, he hasn't 'felt pushed', he is 'not aware', 'may not be able to', whereas GPs who knew the system were a lot more grounded in authoritative mechanisms ('you call X'), like GP3.

GP3 ...(patient) who has a difficult symptom that is too complicated then you call X (local palliative care service).

These perceived difficulties result in less likelihood for GP2 to get expert advice within this system, and the constraints contextually are thus an important factor in the broader cultural framework of out-of-hours services, within which this doctor's sense-making takes place.

GP2 And it does add to those sorts of, one of those things that, are, well, I say this reluctantly, but it, it does sort of, become a bit of a tiresome thing, you know you have to go through, phone through the hospital switchboards, rather than having a quick access number, where you know someone in the local area and the structure was there.

GP2 Very frustrating, yes, it does weigh on your mind and you may be, well again I say this reluctantly but you may feel less inclined to get advice and you know, go with your own knowledge in the first place.

### Language barriers

Information exchange was potentially hampered by more basic aspects of the doctor-patient-carer transaction, such as language barriers and communication skills. GP3 points this out quite politely and laughs, perhaps to lessen the impact of her statement and not appear overly judgemental of some of her foreign colleagues.

*GP3 Then maybe their communication and ****language ****skills (laughs) aren't as good, and so they can't sit down and actually spend the time working out with the family what really they would think should happen.*

Similarly GP5 talks about out-of-hours GPs from different cultures, some of whom he feels are less inclined to ask for help when they might need it. He is also not entirely comfortable talking about this and speaks more silently in the build-up to the comment and repeats the words 'worked with one or two', with longish silences in-between, during which he appears to be thinking about how he should phrase his next words. This is the only time in any of the interviews that language skills or cultural origins are mentioned, both times by British doctors. The brevity of the comments means that not much comment can be made about them specifically, but they are nonetheless very interesting, and could warrant further research.

MT: Do you think that within out-of-hours that there's a few people who might find it difficult to ask for help?

GP4: Yeah (speaks more silently now) (silence 3 seconds)) Worked with one or two. One or two (silence 2 seconds). Most of them are very good, but there's one or two, who think they're the master of everything and, and, and, I think that generally some people come from cultures where, asking for help is seen as a failure somehow. Whereas I think it's, it's a strong thing to do.

### Information exchange between out-of-hours GPs

Another theme was a perceived lack of good information exchange between triage out-of-hours GP (who remain at the out-of-hours base and field phone-calls or see patients there) and mobile out-of-hours GP (who are based in a car and drive to patients' homes).

GP3 But it's a challenge to make sure you, try, if you think something should happen, you try and document that on the computer in the hope that that then goes to the doctor in the car, it's supposed to...

GP7 ...sometime the frustrating part is when, there is some other professional colleagues, they don't always understand, again I can cite an example only recently, where I felt at the end of the phone that this man could have done with a dose of haloperidol to calm him down. You know, while what the GP did, who visited, he ignored my advice he actually increased the dose of diamorphine.

GP7 felt uncomfortable making decisions over the phone in out-of-hours and felt it was unsafe.

*GP7...you ****don't ****(loudly) know the patient, you have not seen the patient, and I find it unsafe doing this.*

### Doctor-nurse communication out-of-hours

There was a division amongst out-of-hours GPs as to their preference on getting information from a nurse, who might be at the patients home and calling them at the out-of-hours base. Some found this very useful.

GP6 ..if you're lucky things have been set-up and planned, some documentation at the house or there's someone on the other side of the phone like a district nurse and then you work something out together.

However, some GPs did not feel they wanted to put their trust in a nurse and base their decision on information over the phone. This was most strongly expressed by GP7.

GP7 You know the difficulty, people will ring, nurses will ring us, they will say, 'Well, can you give us advice or can you authorise dose-change over the end of the phone?', now here you are, you're stuck, you're stuck, because she wants to solve the problem, she's at the sharp end, the nurse there. You don't know her, other than the voice at the end of the line, you have not seen the patient previously, you have not seen the patient now, even to make any judgement, and here you are asked to make a very serious decision on very serious drugs. Which can go wrong, and it they do go wrong, then people can be harmed as well as you can be harmed(...)..a couple of doctors, who they do it, they just sign what the nurse give them, but I don't...

For GP7 this had in the past led to conflict with nurses out-of-hours.

GP7...letting the colleagues down, letting the patient down, conflict with district nurses, having to drag up the issues of safety of the prescribing and the nurses having to come back and re-sort out the, the, you know, second visit by GP or nurse, sort out the syringe-driver or whatever the case may be.

MT: Have you had any problems regarding that, maybe from those nurses who try and get you to sign the drug-chart, without you seeing the patient because they want to get it done?

GP7: Well, I mean, they feel, erm, (silence 2 seconds) they know not to take it personally, they obviously feel maybe that they have been let down, that all they wanted was to get the signature...

Conversely, GP9 felt that nurses often had more knowledge of palliative care than GPs and was happy for them to lead her prescribing decisions and management of palliative care patients.

GP9...I have had some very positive experiences as well, where the nurses have been very useful. And that's been like a hand-holding thing, whilst I've been on-call for out-of-hours.

She uses metaphoric language that equates her to a child that needs support (hand-holding), indicating that she is happy to relinquish control and is confident in the nurses' abilities.

### Accessing pharmacies and medication out-of-hours

All GPs had issues with how to access pharmacies out-of-hours, perceiving this as a major flaw and inconsistency. GP3 uses some candid language in her portrayal of what it is like trying to get medication and syringe-drivers out-of-hours.

GP3 ..and it all becomes a disaster and it's a two or three hour disaster, what with getting medication, getting prescriptions, finding the on-call pharmacist (...) I mean, setting up a syringe-driver can be six or seven or eight phone calls.

And GP2 also recalls problems accessing medicines from on-call pharmacies.

GP2 But I have, I have run into situations where I've felt, that errm, in fact, yes, in fact I had a lot of difficulty getting some M (constipation medicine) for a patient, a patient with bony metastases and it was a Friday evening, (...) If out-of-hours, the pharmacies, they're pretty, some of them are pretty restricted in what they want to make available.

And for GP7, the access to medication was 'all over the place' and he expressed his frustration at this system.

GP7 (...)accessing, getting the medicines is all over the place, you have to phone around and see what and who are stocking which drug.

## Discussion

### Summary of main findings and comparison with existing literature

Information exchange within the out-of-hours system impacted strongly on GPs experiences of palliative care encounters and this was an area GPs felt passionate, some even emotional about, given some of the language used. It did highlight the fact that these out-of-hours GPs felt left alone on occasion, unable to access services and not knowing who to call. The language GPs used to describe these situations expresses their feeling of isolation and anxiety and raises the important question as to whether these concerns are limited to just their palliative care population or whether it extends more broadly, namely to those people who are elderly, frail and in nursing homes.

GPs felt that they were often the ones to pick up the pieces, at night, over weekends or on bank holidays, when it came to palliative care problems. In-hours GPs appeared not to be communicating their palliative care patients with them. There was a desire from out-of-hours GPs, that palliative care patients should have care plans at home which needed regular updating by in-hours and out-of-hours doctors. The data lends support to Burt's audit conducted prior to the new GMS contract [30], which looked at palliative care patient hand-over in GP co-operatives from in-hours to out-of-hours providers. The audit found that the systems in place to alert co-operatives to the needs of palliative care patients were under-utilized and recommended that services move towards an integrated approach encouraging GPs and district nurses to update and transfer information to out-of-hours providers. These findings are similar to those from Worth's study in Scotland also prior to the new GMS contract, where GPs expressed "concern about delivering good palliative care within the constraints of a generic acute service, and problems accessing other health and social care services" [1].

Information exchange systems therefore appear not to have improved since the introduction of the new GMS contract, although comments are limited to the area under investigation for this study, i.e. Cardiff out-of-hours in 2008, only. Also, a study undertaken in 2007 on uptake of end of life care initiatives [31] found comparatively low uptake of the Gold Standards Framework (GSF) in Wales (16%) compared to England (62.4%,) [32]. GSF covers the topic of communication with out-of-hours services, and this perceived lack of communication may be a consequence of Wales' lower uptake of this initiative. In Scotland, a palliative care handover form for NHS24 is being piloted [33]. This form is completed and transmitted electronically via the relevant GP computing software and is to be rolled out in Scotland in the future. This would provide a useful, standardised way of communication that could readily be accessed by telephone out-of-hours responders and could be communicated to the relevant out-of-hours doctors in each locality where necessary.

On the other hand, some out-of-hours doctors sometimes felt low confidence in changing patients' treatment regimes and therefore passed them back on to the in-hours doctors. This caused a degree of guilt and was described as 'passing the buck'.

With regard to available resources, the out-of-hours record keeping system was felt not to be helpful in finding past entries on a particular patient's management, including previous out-of-hours doctor visits. This calls for a review of the entire system, as already proposed by the 2007 scoping exercise on generalist services for adults at the end of life [6]. GPs interviewed were unsure how this should be undertaken, some suggesting hand-held computers for note-keeping, others suggesting notes at palliative care patients' homes. It also raises issues on how to keep patient data confidential, yet still accessible to those who may need it in the out-of-hours setting. A recent qualitative study called for patient-held nursing notes to be completed by all visiting professionals (medical and nursing, in-hours and out-of-hours) [34].

Getting help from palliative care specialists was routine for some, whilst others had no idea how to go about this and signalled discomfort at calling for advice. This lack of knowledge of local advice available from the hospice brings into focus that there is still a need for education and information dissemination locally. Other areas in the United Kingdom have given their system a memorable name, such as the hospice telephone advice service 'Palcall' in Scarborough, [35] and maybe such a branding exercise is useful to bring the service to all out-of-hours doctors' attention.

There was also discernible from interviews a suggestion at potential conflict of doctors with nurses over making prescribing decisions over the phone, based on nurses' recommendations and that doctors did not always feel it appropriate to take advice from nursing colleagues. This hierarchical doctor-nurse interaction has already been noted by another qualitative study in in-hours general practice. It found that implementing the Gold Standards Framework for palliative care in England showed marked variation in how multi-professional co-operation worked in individual teams and that hierarchical doctor-nurse relationships persisted [36].

Given the negative comments made by out-of-hours doctors on access to pharmacies and to medication, it would seem reasonable to look at improving access and/or to adopt an approach of anticipatory prescribing from in-hours providers, as envisaged by the Department of Health in 2005. Their paper "Securing proper access to medicines in the out-of-hours period" called for availability of palliative care medication in patients' homes, organised by in-hours GPs and prescribed on an as-required-basis for nurse administration [37]. However there is likely to be a bias in these interviews, as out-of-hours doctors may only see the proportion of cases where prior planning has not occurred.

There is a need to implement and disseminate palliative care knowledge to the wider general practitioner population, as recommended by the Sugar and Finlay reports. In Wales, a pilot education program for general practitioners on end of life issues organised by palliative care specialists has been rolled out via the Palliative Care Implementation Board for Wales and has seen good uptake [38].

## Conclusions

This interview study explored topics relevant to information exchange and communication within the community out-of-hours setting. Interviewed GPs were at the sharp end of out-of-hours care delivery and felt that there were insufficiencies in the system which made good palliative care delivery difficult. They suggested different strategies on how palliative care could be better communicated between services, which should provide a blueprint for those involved in structuring and improving out-of-hours services as well as primary and specialist palliative care, and the ways in which these services interlink.

### Limitations of study

The study had 9 participants, who came forward after sixty out-of-hours doctors were approached. It is likely that this was a more motivated sub-group, willing to give up their time for this project. On the other hand, this IPA study was not primarily looking for representative statements from GPs, but rather aimed to identify outlying trends and unusual new data within an out-of-hours sub-setting, that, for instance, a postal survey may not have uncovered.

Regarding the sample size of nine it is worth pointing out the approach used in IPA studies; historically, researchers seeking to make an impact in research settings are more familiar with quantitative paradigms and this has meant using larger sample size numbers to 'appease' reviewers for qualitative methodologies [39]. Smith now suggests just 3-6 participants as a reasonable size for an IPA study. This number allows the identification of similarities and differences per group without overwhelming the rich description.

A limitation of this study is the fact that all nine GPs did both in-hours general practice work in addition to out-of-hours work and it would have been interesting to interview GPs who exclusively work out-of-hours. Regarding the study's geographical focus (South Wales), some comments GPs made here may not appear relevant to other areas. It is, however, worth pointing out that many OOH services across the United Kingdom are structured similarly and this could be viewed as a needs assessment relevant to other providers.

The interviews were conducted by a doctor, and this may have had an impact on the answers given by doctor-participants; they may have felt a sense of increased scrutiny or may have been mindful of any limitations with regard to their own palliative care knowledge, leading to less willingness to discuss and explore these aspects fully. This was not noticeable during interviews but may have played a deeper psychological role.

### Implications for future research

The sub-group of out-of-hours doctors who *exclusively *work in this system warrants further exploration, due to their non-exposure to in-hours practice.

Information hand-over systems from in-hours to out-of-hours with regard to palliative care were felt to be inadequate. It would be beneficial to look at areas and practices that are potential centres of excellence in this regard and to try to implement their ideas.

It is also noteworthy that out-of-hours doctors interviewed felt left out of the care loop; none of them had seen any handover sheets from specialist palliative care providers, which is an area where change management could be beneficial. For instance, there could be local discussion as to whether all hospice discharge letters should also be copied to the out-of-hours service.

## Competing interests

The authors declare that they have no competing interests.

## Authors' contributions

MT conducted the interviews and transcribed them. Both authors were involved in the data analysis of transcripts. MT wrote the article and AN made corrections and amendments. Both authors read and approved the final manuscript.

## Pre-publication history

The pre-publication history for this paper can be accessed here:

http://www.biomedcentral.com/1472-684X/9/18/prepub
